# Clinical Evaluation of the Healgen Rapid COVID‐19 Antigen Test as a Point‐of‐Care Diagnostic Tool

**DOI:** 10.1002/iid3.70228

**Published:** 2025-07-29

**Authors:** Stephen A. Young, Hua Zhang, Jose Rodriguez, David Mishkin, Ward Paine, Li Seyfried, LaTisha Hargrove, Dennis L. Broyles, Chermaen Lindberg, Anurag Purushothaman, Stacey House

**Affiliations:** ^1^ TriCore Reference Laboratories Albuquerque New Mexico USA; ^2^ Zhejiang Orient Gene Biotech Co. Ltd. Zhejiang China; ^3^ Golden Research Miami Florida USA; ^4^ Proactive Clinical Research Fort Lauderdale Florida USA; ^5^ Frontier Clinical Research LLC Morgantown West Virginia USA; ^6^ Healgen Scientific LLC Houston Texas USA; ^7^ CovarsaDx Downey California USA; ^8^ Department of Emergency Medicine Washington University School of Medicine St. Louis Missouri USA

**Keywords:** COVID‐19, Healgen rapid COVID‐19 antigen test, point‐of‐care, SARS‐CoV‐2

## Abstract

**Background:**

Development of readily available Rapid COVID‐19 Antigen tests essential for promptly identifying SARS‐CoV2 infection and preventing its spread.

**Methods:**

This study evaluated the clinical performance of the Healgen Rapid COVID‐19 antigen test as a point‐of‐care diagnostic tool with 806 evaluable subjects who were within 6 days post‐symptom onset. The results from the Healgen test were compared to the results from Emergency Use Authorization (EUA) approved SARS‐CoV‐2 RT‐PCR tests.

**Results:**

Out of the 806 evaluable subjects, 140 tested positive and 640 tested negative for SARS‐CoV‐2 with the Healgen COVID‐19 test, showing good agreement with the EUA RT‐PCR results. There were 26 subjects with discordant results, of which 24 were negative according to the Healgen test but positive according to the RT‐PCR test, while 2 were positive by the Healgen test but negative by the EUA‐PCR test. The positive percent agreement (PPA) and negative percent agreement (NPA) were 85.4% and 99.7%, respectively. Additionally, the Healgen COVID‐19 test detected 34 cases (60.7%) out of 56 weak positive cases (based on Ct values of ≥ 30 by the EUA PCR test), demonstrating good detection capability of the test.

**Conclusions:**

The Healgen Rapid COVID‐19 antigen detection test demonstrated good performance in terms of PPA and NPA when compared to the EUA RT‐PCR assays and has potential as a diagnostic tool for SARS‐CoV‐2.

## 1 Introduction

The coronavirus disease 2019 (COVID‐19) pandemic represents one of the foremost global health crisis and significant health challenges of our time. By 2020, antigen rapid tests gained approval from regulatory bodies worldwide for COVID‐19 testing. Point‐of‐care (POC) rapid antigen tests, such as a lateral flow immunochromatographic‐based tests, have emerged as an important and routine diagnostic tool for COVID‐19 [1, 2]. These POC rapid antigen tests hold great promise for expanding testing accessibility, accelerating infection detection, guiding clinical and public health management decisions aimed at curtailing transmission, and for enabling timely treatment with antiviral drugs. Moreover, rapid antigen detection diagnostic tests require considerably less time and can be performed with ease compared to the high complexity laboratory RT‐PCR tests for SARS CoV‐2.

The primary approach for early diagnosis of SARS‐CoV‐2 infection and prompt isolation typically involves testing individuals exhibiting symptoms or with confirmed exposure to infected individuals [3]. Since the beginning of the SARS‐CoV‐2 pandemic, various prominent variants of the virus, such as Alpha, Beta, Delta, and Omicron, emerged and exhibited enhanced transmission, severity, and immune evasion capabilities [4]. Development of reliable rapid antigen detection tests with high positive percent agreement (PPA) and negative percent agreement (NPA) is therefore essential for prompt and accurate diagnosis of COVID‐19, as well as for making tailored medical management decisions, which in turn may aid in mitigating the spread of the virus.

This study aimed to determine the diagnostic PPA and NPA of Healgen's newly developed Rapid COVID‐19 Antigen Test, a lateral flow immunochromatographic assay designed for the qualitative detection of SARS‐CoV‐2 nucleocapsid protein in direct anterior nasal swab specimens from symptomatic individuals within 6 days of symptom onset. The study was a prospective performance evaluation of the Healgen Rapid COVID‐19 Antigen Test, compared to results from highly sensitive EUA RT‐PCR tests, including the EUA Cobas SARS‐CoV‐2 & Influenza A/B Test on the Roche Cobas 6800, the EUA Cepheid Xpert Xpress SARS‐CoV‐2/Flu/RSV Plus, and the BioFire Respiratory Panel 2.1 (RP2.1). Overall, after analyzing 806 evaluable subjects, the Healgen Rapid COVID‐19 test demonstrated robust agreement with the comparator tests, with a PPA of 85.4% and an NPA of 99.7%.

## 2 Materials and Methods

### 2.1 Patients

This prospective study enrolled 867 subjects (aged 2 and older) at six clinical sites in the United States. Sample collection was performed between May and July of 2022. Demographics of each subject were recorded. Inclusion criteria were male or female subjects 2 years or older, subjects exhibiting fever, or two or more symptoms associated with COVID‐19 according to the Centers for Disease Control and Prevention (CDC) (such as, but not limited to, chills, cough, shortness of breath or difficulty breathing, fatigue, muscle or body aches, headache, new loss of taste or smell, sore throat, congestion or runny nose, nausea or vomiting, or diarrhea) who presented within 7 days of symptom onset, or subjects without any signs or symptoms associated with COVID‐19 in the last 14 days. Subjects not able or willing to contribute the required swab samples for testing, who did not complete the study procedures, or subjects not willing to sign the study informed consent were excluded from the study. The study was approved by the WCG IRB, and all subjects provided written informed consent.

### 2.2 Study Design

The primary objective of the study was to evaluate the clinical performance (PPA and NPA) of the Healgen Rapid COVID‐19 Antigen Test using anterior nasal samples collected from both nostrils, either by participants themselves, by another individual under healthcare professional supervision, or directly by healthcare professionals. Subsequently, the samples were tested immediately by a healthcare professional following the instructions for use. Additionally, a nasopharyngeal sample from each subject was collected (from one nostril only) by a healthcare professional for the comparator testing using the following EUA RT‐PCR tests: such as the EUA cobas SARS‐CoV‐2 and Influenza A/B Test on the Roche cobas 6800, the EUA SARS‐CoV‐2/Flu/RSV Plus on the Cepheid Xpert Xpress, and the BioFire Respiratory Panel 2.1 (RP2.1) on the bioMérieux. Briefly, the comparator nasopharyngeal samples were placed in either 3 mL UTM (BD UVT) and refrigerated at 2°C to 8°C, or at −70°C or colder, before shipping to the TriCore Reference Laboratories (Albuquerque, New Mexico) for comparator testing.

### 2.3 Investigational Product

The Healgen Rapid COVID‐19 Antigen Test is a lateral flow immunochromatographic assay that uses colloidal gold‐conjugated monoclonal antibodies to detect the SARS‐CoV‐2 nucleocapsid protein in anterior nasal swab specimens from symptomatic individuals. These specimens can be collected by a healthcare provider (HCP), or self‐collected (by a parent or guardian for children under 14, or by individuals aged 14–17 under the supervision of an adult or HCP). The use of Rapid COVID‐19 Antigen Test testing is restricted to laboratories certified under the Clinical Laboratory Improvement Amendments of 1988 (CLIA), 42 U.S.C. §263a, that meet the requirements to perform high complexity, moderate complexity, or waived tests.

### 2.4 Statistical Analysis

All data were analyzed using the R statistical software, version 4.1. Diagnostic test performance metrics were estimated using the Hmisc package in R. All clinical data recorded in the electronic data capture system were 100% source data verified. Demographics were tabulated based on age, sex, race, and ethnicity (Table 1), as was the number of evaluable subjects by age group by site (Table 2). Results were calculated for positive percent agreement (PPA), negative percent agreement (NPA), positive predictive value (PPV), and negative predictive value (NPV) using data from the 2 × 2 table (Table 3). PPA was also calculated separately for each site (Table 4), as well as by age group and days post‐symptom onset (Table 8).

**Table 1:** Demographics of subjects participated in the study.

Age of subject (years)	Mean	SD
	40.5	19.6
	Median	Range
	39.0	2.0–93.0
Subject sex at birth	Total No. (%)	
Female	467 (57.9%)	
Male	339 (42.1%)	
Subject ethnicity:	Total No. (%)	
Hispanic/Latino	485 (60.2%)	
Not Hispanic/Latino	320 (39.7%)	
Unknown/Prefer not to answer	1 (0.1%)	
Subject race	Total No. (%* **)**	
American Indian or Alaskan Native	0 (0%)	
Asian	11 (1.4%)	
Black/African American	140 (17.4%)	
Native Hawaiian or Pacific Islander	0 (0%)	
White	644 (79.9%)	
Unknown/prefer not to answer	0 (0%)	
Other	11 (1.4%)	

* Total = 100.1% due to rounding.

**Table 2:** Evaluable subjects by age group by site.

Sites	2–< 14 years of age	14–24 years of age	> 24–64 years of age	≥ 65 years of age
Golden Research, Florida	44	48	262	45
Washington University School of Medicine, Missouri	0	7	67	21
Proactive Clinical Research—Sarasota, Florida	5	8	52	7
University of Michigan, Ann Arbor	0	14	30	2
Proactive Clinical Research—Fort Lauderdale, Florida	2	4	14	8
Frontier Clinical Research—Morgantown, West Virginia	24	37	89	16
Total	75	118	514	99

**Table 3:** Comparative performance analysis of the Healgen rapid COVID‐19 antigen test vs. composite EUA RT‐PCR tests.

Healgen COVID‐19 test (symptomatic subjects within 6 DPSO)	Composite comparator		
	Positive	Negative	Total
HCP collected samples			
Positive	71	1	72
Negative	11	322	333
Self/lay user collected samples			
Positive	69	1	70
Negative	13	318	331
Total	164	642	806

*Note:* Positive Percent Agreement (PPA) = (71 + 69/164) = 85.4% (95% CI: 79.1%–90%).

Negative Percent Agreement (NPA) = (322 + 318/642) = 99.7% (95% CI: 98.9%–99.9%).

Positive Predictive Value (PPV) = (140/142) × 100 = 98.6%.

Negative Predictive Value (NPV) = (640/664) × 100 = 96.4%.

**Table 4:** Clinical performance evaluation of the Healgen rapid COVID‐19 antigen test by site.

Testing site name	No. of subjects tested	Investigational positives	Comparator positives	% Positive rate (by comparator)	PPA (95% CI)
Golden Research	399	64	76	19.0%	84.2% (74.4%–90.7%)
Washington University School of Medicine	95	20	23	24.2%	87.0% (67.9%–95.5%)
Proactive Clinical Research Sarasota	72	7	9	12.5%	77.8% (45.3%–93.7%)
University of Michigan	46	13	15	32.6%	86.7% (62.1%–96.3%)
Proactive Clinical Research Fort Lauderdale	28	8	10	35.7%	80.0% (49.0%– 94.3%)
Frontier Clinical Research Morgantown	166	28	31	18.7%	90.3% (75.1%–96.7%)
Total	806	140	164	20.3%	85.4% (79.1%–90.0%)

The agreement between the Healgen Rapid COVID Antigen Test and the EUA RT‐PCR was calculated in symptomatic subjects with 95% confidence intervals.

## 3 Results

### 3.1 Patient Population Characteristics

In this prospective study, a total of 867 subjects aged 2 and above were enrolled. Of the 867 subjects enrolled, there were 806 evaluable subjects (339 male and 467 females with a median age of 39 years), who were within 6 days post‐symptom onset (DPSO), and 61 unevaluable subjects. Although the initial inclusion criteria for the study covered subjects within 7 days of symptom onset, performance evaluation was restricted to subjects within 6 days of symptom onset based on FDA's clinical data evaluation and recommendations. As a result, 17 subjects who exhibited symptoms beyond 6 days post‐symptom onset were excluded from the analysis. Of the 806 evaluable subjects, 405 were collected by HCP, and 401 were either self‐collected or collected by lay users. Among the 61 unevaluable subjects, 24 were excluded per protocol for the primary objective, 20 were asymptomatic, and 17 had symptoms occurring more than 6 days post‐symptom onset. The baseline demographics of evaluable subjects and their enrollment across different age groups at each site are detailed in Tables 1 and 2.

### 3.2 Overall Clinical Performance of Healgen Rapid COVID‐19 Antigen Test

Among the 806 evaluable subjects, 164 tested positive based on composite comparator testing, and the Healgen Rapid COVID‐19 Test accurately detected 140 positive cases among them (Table 3). All samples were tested using two comparator RT‐PCR assays (EUA cobas SARS‐CoV‐2 & Influenza A/B Test on the Roche cobas 6800 and the EUA SARS‐CoV‐2/Flu/RSV Plus on the Cepheid Xpert Xpress), and if the results of these assays did not agree, the BioFire Respiratory Panel 2.1 (RP2.1) on the bioMérieux was used as the third comparator assay to serve as the tie‐breaker. There were 24 cases with discordant results due to a false negative with the Healgen Rapid COVID‐19 Antigen Test. The overall results showed a positive percent agreement (PPA) of 85.4% (95% CI: 79.1%–90.0%), highlighting a strong concordance between the Healgen Rapid COVID‐19 Antigen Test and the composite results from the comparator RT‐PCR tests in identifying positive SARS‐CoV‐2 cases (Table 3). Likewise, the Healgen rapid COVID‐19 rapid test identified 640 negative cases out of 642 cases that tested negative by the composite comparator method (Table 3). Two cases were discordant due to a false positive by the Healgen Rapid COVID‐19 Antigen test. Overall, these results showed a negative percent agreement (NPA) of 99.7% (95% CI: 98.9%–99.9%) (Table 3), further underscoring a robust concordance between the Healgen Rapid COVID‐19 Antigen Test and RT‐PCR. In addition, the test demonstrated a positive predictive value (PPV) of 98.6% and a negative predictive value (NPV) of 96.4%, further supporting its diagnostic utility in identifying SARS‐CoV2 infection. While RT‐PCR is widely accepted as the reference standard for SARS‐CoV‐2 detection, it has limitations and may produce false results, particularly in cases with low viral load. As a result, performance metrics such as PPA, NPA, PPV, and NPV are subject to potential misclassification bias. These values should be interpreted with consideration of the validity of the reference test, particularly in varying prevalence settings. The performance evaluation of the Healgen Rapid COVID‐19 Antigen Test at each site is shown in Table 4.

After further analysis of the comparator RT‐PCR Ct values, using a low positive Ct cutoff of 30 [5], there were 56 (34.1%) out of the 164 positive cases that were low positive samples with Ct values ≥ 30 based on the Target 2 (pan‐Sarbecovirus) gene. Importantly, the Healgen Rapid COVID‐19 Antigen Test was able to detect 34 (60.7%) of the 56 low‐positive samples, highlighting its capability in detecting SARS‐CoV‐2 even at lower viral loads. Analytical studies (data not shown) confirmed that the performance of the test was not impacted by specimens not tested immediately after collection and that specimens were stable up to 4 h at room temperature after collection and before testing.

In addition, inclusivity testing with currently available commercial stock strains of SARS‐CoV‐2 (alpha, beta and delta variants were obtained from BEI; and gamma, kappa and omicron variants from ZeptoMetrix) identified that the test device could detect different variants of SARS‐CoV‐2 such as the alpha (B.1.1.7), beta (B.1.351), delta (B.1.617.2), gamma (P1), kappa (B.1.617.1), and omicron (B.1.1.529) variants with lowest detectable concentrations (LoD) at 1 × 10^2^, 3.83 × 10^2^, 1.1 × 10^2^, 6.3 × 10^2^, 1.9 × 10^2^, and 2.51 × 10^2^ TCID_50_/mL, respectively (Table 5). Moreover, a high dose‐hook effect study to evaluate the occurrence of false negative test results in the presence of very high levels of target demonstrated no high dose hook effect for concentrations up to 5.75 × 10^6^ TCID_50_/mL of heat‐inactivated SARS‐CoV‐2 (USA‐WA 1/2020). The lowest concentration of SARS‐CoV‐2 (USA‐WA 1/2020) (limit of detection) that the Healgen Rapid COVID‐19 Antigen Test can detect 95% of the time was determined as 5.75 × 10^3^ TCID_50_/mL.

**Table 5:** Inclusivity testing using different variants of SARS‐CoV‐2.

SARS‐CoV‐2 variant	Sublineage	Lowest concentration tested positive for all 3 replicates in 3 lots (TCID_50_/mL)
Delta	B.1.617.2	1.10 × 10^2^
Beta	B.1.351	3.83 × 10^2^
Alpha	B.1.1.7	1.0 × 10^2^
Omicron	B.1.1.529	2.51 × 10^2^
Gamma	P1	6.30 × 10^2^
Kappa	B.1.617.1	1.90 × 10^2^

### 3.3 Analysis of Cross Reactivity, Microbial Interference, and Endogenous Substance Interference With the Healgen Rapid COVID‐19 Antigen Test

Microbial cross‐reactivity and interference studies were performed to determine whether microorganisms that might be present in respiratory samples would cross‐react or interfere with device performance. The concentrations for the various microorganisms and endogenous substances were determined based on the recommendations from the FDA's SARS‐CoV‐2 Antigen Template for Test Developers. Three replicates of each 1:1 dilution of microorganism stock prepared in negative matrix were tested in the absence and presence of 2x LoD inactivated SARS‐CoV‐2 virus (USA‐WA1/2020). Three lots of the Healgen Rapid COVID‐19 antigen test kits were tested in this challenge. Fifty (50) μL of each prepared sample was applied to kit swabs and eluted into the extraction buffer tube. No cross‐reactivity or microbial interference was observed with the organisms tested (Table 6).

**Table 6:** Microorganisms tested for cross‐reactivity and interferences.

Microorganism	Concentration tested	Cross‐reactivity	Interference
Human coronavirus 229E	8.00 × 10^5^ TCID_50_/mL	No	No
Human coronavirus OC43	7.00 × 10^6^ TCID_50_/mL	No	No
Human coronavirus NL63	2.93 × 10^4^ TCID_50_/mL	No	No
MERS‐coronavirus	7.0 × 10^5^ TCID_50_/mL	No	No
Adenovirus 21	2.39 × 10^6^ TCID_50_/mL	No	No
Adenovirus 10	1.14 × 10^6^ TCID_50_/mL	No	No
Human Metapneumovirus	3.95 × 10^5^ TCID_50_/mL	No	No
Parainfluenza virus Type 1	2.23 × 10^6^ TCID_50_/mL	No	No
Parainfluenza virus Type 2	2.23 × 10^5^ TCID_50_/mL	No	No
Parainfluenza virus Type 3	4.00 × 10^6^ TCID_50_/mL	No	No
Parainfluenza virus Type 4a	7.0 × 10^4^ TCID_50_/mL	No	No
Influenza virus, Type A (H1N1)	4.0 × 10^8^ CEID_50_/mL	No	No
Influenza virus, Type A (H3N2)	7.00 × 10^5^ TCID_50_/mL	No	No
Influenza virus, Type B	7.00 × 10^5^ TCID_50_/mL	No	No
Enterovirus 68	2.23 × 10^6^ TCID_50_/mL	No	No
Enterovirus 71	4.00 × 10^7^ TCID_50_/mL	No	No
Respiratory syncytial virus	2.23 × 10^6^ TCID_50_/mL	No	No
Rhinovirus 60	8.00 × 10^5^ TCID_50_/mL	No	No
*Haemophilus influenzae*	1.74 × 10^8^ CFU/mL	No	No
*Streptococcus pneumoniae*	3.35 × 10^8^ CFU/mL	No	No
*Streptococcus pyogenes*	5.98 × 10^8^ CFU/mL	No	No
*Candida albicans*	1.19 × 10^8^ CFU/mL	No	No
*Bordetella pertussis*	4.90 × 10^9^ CFU/mL	No	No
*Mycoplasma pneumoniae*	6.75 × 10^7^ CCU/mL	No	No
*Chlamydia pneumoniae*	4.25 × 10^7^ CFU/mL	No	No
*Legionella pneumophila*	9.20 × 10^9^ CFU/mL	No	No
*Staphylococcus aureus*	5.0 × 10^6^ CFU/mL	No	No
*Staphylococcus epidermidis*	1.75 × 10^8^ CFU/mL	No	No
Pooled human nasal wash	N/A	No	No

Furthermore, in‐silico analysis was conducted for several pathogens including SARS‐coronavirus, Human coronavirus HKU1, *Mycobacterium tuberculosis*, *Pneumocystis jirovecii* (PJP), and for comparison, MERS coronavirus. The N protein sequence (GenBank ID: UUL70282.1) from an Omicron variant of SARS‐CoV‐2, encompassing sub‐lineages BA.1, BA.1.1, BA.2 (Accession Number OP160218), served as the reference for the analysis. Results indicated that there was no significant sequence homology with this N protein sequence in either *Mycobacterium tuberculosis* or *Pneumocystis jirovecii* genomes, suggesting the absence of cross‐reactivity.

Homologous sequences (N proteins) were identified in SARS‐coronavirus, Human coronavirus HKU1, and MERS coronavirus. The N protein of Human coronavirus HKU1 shares 41.18% to 49.00% homology or identity with the SARS‐CoV‐2 Omicron N protein sequence, while that of MERS coronavirus shares 52.87% to 54.04% identity. Although the homology is relatively low, the possibility of cross‐reactivity cannot be ruled out. The N protein sequence of SARS coronavirus shares 79.01% to 97.61% sequence identity with that of the SARS‐CoV‐2 Omicron variant, indicating a likelihood of cross‐reactivity. Since cross‐reactivity with SARS‐CoV, MERS‐CoV, and HKU1 cannot be ruled out for SARS‐CoV‐2, it is stated in the intended use that it cannot distinguish between SARS‐CoV‐2 and SARS‐CoV.

Potentially interfering substances that might be present in respiratory samples were tested to determine if interference would occur on the Healgen Rapid COVID‐19 Antigen Test. The potential interfering substances were tested with negative samples and low positive samples (2x LoD) SARS‐CoV‐2 (USA‐WA1/2020) in triplicate, with three lots of tests. Results showed that none of the tested endogenous/exogenous substances interfered with the test at the concentrations examined (Table 7).

**Table 7:** Potential interfering substances for respiratory samples.

Substance	Concentration	Cross‐reactivity	Interference
Whole blood	4%	No	No
Human leukocytes	1 × 10^7^ cells/mL	No	No
Mucin	0.5%	No	No
Chloraseptic (menthol/benzocaine)	3 mg/mL	No	No
Naso GEL (NeilMed)	5% v/v	No	No
CVS nasal drops (phenylephrine)	15% v/v	No	No
Afrin (oxymetazoline)	15% v/v	No	No
CVS nasal spray (cromolyn)	15% v/v	No	No
Zicam	5% v/v	No	No
Homeopathic (alkalol)	15%	No	No
Sore throat phenol spray	15% v/v	No	No
Hand soap	1%	No	No
Hand sanitizer	1%	No	No
Tobramycin	4 μg/mL	No	No
Mupirocin	10 mg/mL	No	No
Fluticasone propionate	15% v/v	No	No
Tamiflu (oseltamivir phosphate)	5 mg/mL	No	No

### 3.4 Clinical Performance Evaluation of the Healgen Rapid COVID‐19 Antigen Test Across Various Age Groups and Days Post Symptom Onset (DPSO)

The testing results of the Healgen Rapid COVID‐19 Antigen test across different age groups and different DPSOs are summarized in Table 8. For age groups 2 to < 14 years and 14–24 years, the PPA between the comparator RT‐PCR and Healgen Rapid COVID‐19 Antigen Test was 84.6% and 89.5% respectively. Consistently, the PPA between the results obtained from the two tests for age groups > 24–64 years and ≥ 65 years were 82.7% and 95.5% respectively (Table 8). Two subjects that showed false‐positive results by Healgen's investigational test were excluded from the investigational positives in the PPA analysis.

**Table 8:** Comparative performance analysis of the Healgen rapid COVID‐19 antigen test vs. composite EUA RT‐PCR tests by age and days post‐symptom onset (DPSO).

Subject age	No. of subjects tested	Investigational positives	Comparator positives	% Positive rate (by comparator)	PPA (95% CI)
2–< 14 years	75	11	13	17.3%	84.6% (57.8%–95.7%)
14–24 years	118	17	19	16.1%	89.5% (68.6%–97.1%)
> 24–64 years	514	91	110	21.4%	82.7% (74.6%–88.7%)
≥ 65 years	99	21	22	22.2%	95.5% (78.2%– 99.8%)
Total	806	140	164	20.3%	85.4% (79.1%–90.0%)
DPSO					
0	26	3	3	11.5%	100.0% (43.9%–100.0%)
1	92	14	16	17.4%	87.5% (64.0%–96.5%)
2	213	36	46	21.6%	78.3% (64.4%–87.7%)
3	219	31	39	17.8%	79.5% (64.5%– 89.2%)
4	150	27	29	19.3%	93.1% (78.0%–98.1%)
5	75	17	19	25.3%	89.5% (68.6%–97.1%)
6	31	12	12	38.7%	100.0% (75.8%–100.0%)
Total	806	140	164	20.3%	85.4% (79.1%–90.0%)

The results for the Healgen Rapid COVID‐19 Antigen Test based on days post‐symptom onset are shown in Table 8. For subjects within the first 6 days of symptom onset, the Healgen Rapid COVID‐19 Antigen test demonstrated a positive agreement of 85.4% compared to the EUA RT‐PCR results. The PPA between the Healgen Rapid COVID‐19 Antigen Test and the EUA RT‐PCR results for 0–6 days post symptom onset was 100%, 87.5%, 78.3%, 79.5%, 93.1%, 89.5%, and 100%, respectively (Table 8).

## 4 Discussion

In this study, the Healgen Rapid COVID‐19 Antigen Test was evaluated for detecting the nucleocapsid protein of SARS‐CoV‐2 from nasal swab collected specimens. The Healgen Rapid COVID‐19 Antigen Test was recently granted FDA 510(k) clearance for point‐of‐care (POC) use, with a moderate complexity level, in diagnosing COVID‐19 among individuals aged 2 and above and within the first 6 days of symptom onset. The application for a CLIA waiver was approved, allowing its use in patient care settings that operate under a CLIA Certificate of Waiver, Certificate of Compliance, or Certificate of Accreditation. In this prospective multi‐center clinical study, 806 evaluable subjects within the first 6 days of COVID‐19 symptom onset were enrolled, resulting in a PPA of 85.4% between the Healgen Rapid COVID‐19 Antigen Test and the comparator RT‐PCR tests.

The Healgen Rapid COVID‐19 Antigen Test addresses the pressing demand for rapid tests to diagnose COVID‐19. The high sensitivity of the antibodies used in the test device was also evident from the ability to detect 60.7% of the weak positive COVID‐19 cases. It should be noted that false negative results might still occur with the Healgen Rapid COVID‐19 Antigen Test. One potential factor could be the limited sensitivity of rapid tests in detecting weak positive cases compared to a molecular RT‐PCR test. When the virus concentration is low or below the LoD, the test can sometimes show false negative results. Additionally, in some cases, determining the exact start date of SARS‐CoV‐2 infection (symptom onset) or the duration of infection in a patient can be challenging. Consequently, at the time of testing, the antigen levels may be below the LoD of the device. Another reason could be the difference in samples used for the Healgen Rapid COVID‐19 Antigen Test and those used for the RT‐PCR. While the anterior nares (AN) swab is used for the Healgen Rapid COVID‐19 Antigen Test, nasopharyngeal (NP) swabs were used for RT‐PCR testing. NP swabs are considered the reference standard for diagnosing SARS‐CoV‐2 infections and are more sensitive than AN swabs [6]. Therefore, negative test results in rapid tests in symptomatic individuals should be considered presumptive, highlighting the importance of confirming these results with a molecular test for SARS‐CoV‐2.

The comprehensive detection capability of the Healgen Rapid COVID‐19 Antigen Test to detect six COVID‐19 variants (alpha, beta, delta, omicron, gamma, and kappa) underscores its robustness and effectiveness in identifying the genetically diverse SARS‐CoV‐2 strains. This is crucial for effective surveillance and monitoring of COVID‐19 outbreaks, especially considering the dynamic nature of the virus and the emergence of new variants over time. For example, in November 2021, the WHO Technical Advisory Group on SARS‐COV‐2 Virus Evolution classified Omicron (B.1.1.529) as a SARS‐CoV‐2 variant of concern [7]. The inclusion of the Omicron variant in the test's detection capability suggests that the Healgen Rapid COVID‐19 Antigen Test could serve as a valuable tool for epidemiological studies, clinical diagnosis, and public health interventions aimed at controlling the spread of COVID‐19. During the clinical trial study period, the predominant circulating variant was Omicron and its subvariants, further supporting the relevance of the study findings in real‐world settings. However, the lack of variant analysis in the clinical study samples is a limitation of this study. Given that Omicron is considered more contagious than previous variants of concern, and considering the potential emergence of new, future variants of concern, there should be an immediate reassessment of the performance characteristics of all rapid antigen tests [8].

Rapid antigen tests demand significantly lower technical expertise and laboratory capacity [9]. As point‐of‐care devices, these tests can be performed by individuals with minimal training across diverse primary and community settings [3]. Therefore, rapid tests offer several advantages: they save time, eliminate the need for specialized equipment, are simple to perform, and require only minimal training. The test can be conducted at the bedside, at any clinic or laboratory, as well as at airports or railway stations [10]. While we acknowledge that the Healgen Rapid COVID‐19 Antigen Test does not introduce novel methodological advancements in the field and operates on the same lateral flow immunochromatographic principle as other rapid antigen tests, its practical advantages make it a valuable addition to the existing landscape of SARS‐CoV‐2 diagnostics. The test offers a user‐friendly design, enabling individuals with minimal training to conduct testing in primary and community healthcare settings. Unlike other SARS‐CoV‐2 point‐of‐care tests that require additional instrumentation for result interpretation, this test is visually read, eliminating the need for specialized equipment and streamlining the testing process. This enhances accessibility, reduces operational complexity, and makes it a more efficient option for widespread deployment, offering meaningful contributions to improving COVID‐19 testing.

A critical concern with most rapid diagnostic tests is the impact on specificity posed by potential cross‐reactivity with other microorganisms or interfering substances [11, 12, 13, 14, 15, 16, 17]. The World Health Organization (WHO) has listed malaria and dengue among the organisms to be tested for cross‐reactivity of COVID‐19 serological tests [18]. A recent study examining a COVID‐19 antibody‐detecting rapid diagnostic test revealed significant cross‐reactivity when challenged with pre‐pandemic malaria, schistosomiasis, and dengue samples [19]. The Healgen Rapid COVID‐19 Antigen Test underwent rigorous analytical evaluation for potential cross‐reactivity with other microorganisms and substances commonly found in respiratory samples, and which could potentially affect test performance. Notably, no instances of cross reactivity were observed across the 28 different microorganisms tested, including human coronaviruses 229E, NL63, and OC43, as well as Influenza A and B. Furthermore, none of the 17 substances possibly present in respiratory samples, ranging from blood components to various medications, exhibited any cross‐reactivity or interference with this test. These findings suggest that the assay maintains high selectivity for detecting the SARS‐CoV‐2 antigen in respiratory samples, even in the presence of commonly encountered substances. Product engineering can optimize specificity [20], and results from the cross‐reactivity studies done underscore the robustness and reliability of this test in real‐world clinical settings.

The possibility of cross‐reactivity of the Healgen RAPID COVID‐19 Antigen test with other human coronaviruses such as SARS, HKU1, and MERS, cannot be ruled out due to their sequence similarity with SARS‐CoV2. Given the high similarity between the N‐proteins of SARS‐CoV2 and SARS‐CoV, these viruses share common antigenic epitopes than other coronaviruses [21]. Patient‐derived monoclonal antibodies to SARS‐CoV‐2 nucleocapsid protein N‐terminal and C‐terminal domains have shown cross reactivity with their counterparts of SARS‐CoV, but not with other human beta‐coronaviruses, highlighting a high degree of amino acid sequence homology between these two viruses [21]. Additionally, serum antibodies from recovered SARS‐CoV patients and immunized animals have demonstrated cross‐reactive neutralization of SARS‐CoV2, further supporting sequence similarity [22]. Moreover, due to the high level of similarity in amino acid sequence, many N antigen detecting lateral flow immunoassays do not differentiate between SARS‐CoV and SARS‐CoV‐2 [23]. While theoretical cross‐reactivity remains a consideration, the low global prevalence of SARS‐CoV and MERS‐CoV reduces the likelihood of misclassification in clinical settings. This limited prevalence minimizes the potential impact of cross‐reactivity on diagnostic performance. Furthermore, the distinct epidemiological patterns of HKU1 and other seasonal coronaviruses, along with the typically mild nature of the diseases they cause, suggest that their impact on test interpretation is negligible [24].

Although clinical validation was assessed prospectively for diagnostic performance and usability in real‐life setting of the target population, the study has its limitations. The main limitation of the current study is the lack of clinical performance evaluation of the Healgen Rapid COVID‐19 Antigen Test in pediatrics below 2 years of age. This is important because data on the performance of SARS‐CoV‐2 rapid antigen detection assays at POC in children are scarce and divergent to some extent [25, 26, 27]. Furthermore, the study was conducted only in symptomatic participants and not in asymptomatic participants. In asymptomatic individuals, the sensitivity of rapid antigen tests is lower and less effective, primarily due to reduced viral shedding, which increases the likelihood of false‐negative results [28]. A study by Soni et al. [29] emphasized the importance of serial testing, particularly in asymptomatic individuals, to enhance detection sensitivity. False‐negative results can have significant public health implications. Asymptomatic individuals who receive a false‐negative result may unknowingly contribute to viral transmission, particularly in high‐risk environments such as healthcare facilities, schools, and workplaces. This highlights the need for repeated testing and confirmatory molecular testing when clinically indicated. Public health strategies should emphasize that a negative antigen test does not rule out infection, and symptomatic or high‐risk individuals should continue to follow appropriate precautions even if they test negative. The strength of the study is that it provides a practical assessment of the device's performance in the POC setting.

The primary care diagnostic process depends on accurate, reliable, and reproducible laboratory testing [30]. Point‐of‐care diagnostic testing aims at reducing the time required to obtain test results, facilitating rapid clinical decision‐making for both clinicians and patients. However, given their possible use in settings with limited resources, the benefits of point‐of‐care tests must outweigh their costs [31]. Therefore, rigorous evaluations of sensitivity and specificity are imperative to ensure that test results can effectively guide treatment decisions in resource‐constrained environments [31, 32]. The excellent diagnostic performance of Healgen's Rapid COVID‐19 Antigen Test represents a significant advancement in the rapid detection of COVID‐19. Its low complexity, speed, and affordability make it an ideal tool for point‐of‐care testing, particularly in settings where molecular testing is limited. By expanding access to a wider range of communities the Healgen's Rapid COVID‐19 Antigen Test can significantly improve COVID‐19 management, especially in situations where rapid diagnosis is essential.

## Author Contributions

**Stephen A. Young:** investigation. **Hua Zhang:** investigation and methodology. **Jose Rodriguez:** investigation. **David Mishkin:** investigation. **Ward Paine:** investigation. **Li Seyfried:** methodology, project administration, and supervision. **LaTisha Hargrove:** project administration and supervision. **Dennis L. Broyles:** methodology and validation. **Chermaen Lindberg:** methodology and validation. **Anurag Purushothaman:** validation, writing, and reviewing. **Stacey House:** investigation.

## Conflicts of Interest

The authors declare no conflicts of interest.

## Data Availability

The clinical data that support the findings of this study are available upon reasonable request.
